# Smaller Cigarette Pack as a Commitment to Smoke Less? Insights from Behavioral Economics

**DOI:** 10.1371/journal.pone.0137520

**Published:** 2015-09-10

**Authors:** Joachim Marti, Jody Sindelar

**Affiliations:** 1 Academic Unit of Health Economics, Leeds Institute of Health Sciences, University of Leeds, Leeds, United Kingdom; 2 Department of Health Policy and Management, Yale School of Public Health, Yale University, New Haven, United States of America; Institutes for Behavior Resources and Johns Hopkins University School of Medicine, UNITED STATES

## Abstract

Cigarettes are commonly sold in packs of 20 units and therefore little is known about the potential impact of pack size on consumption. Using insights from behavioral economics, we suggest that cigarette packs smaller than the standard size may help some smokers cut back and/or quit, consistent with their long-term goals. Results from an online hypothetical purchase experiment conducted in a sample of US smokers reveal that over a third of smokers are willing to pay a price premium to purchase in smaller quantities. Further, a desire to quit smoking and high self-control is associated with preference for a smaller pack. While we provide some preliminary evidence that smaller packs may be beneficial to certain types of smokers, further research should be conducted to assess whether the smaller pack size should be considered in the arsenal of tobacco control policies to help current smokers quit (JEL: I18; I12; D12)

## Introduction

Policy-makers are increasingly concerned with cigarette packaging as it can be used by tobacco companies to entice smokers and by governments to provide textual and graphic health warnings [1–3]. The World Health Organization Framework Convention on Tobacco Control (WHO FCTC) includes guidelines on tobacco packaging (i.e. shape, size, and color), as the pack has become one of the few remaining marketing tools available to the tobacco industry [4, 5]. Australia implemented the first plain packaging regulations in 2012 (i.e. no brand, no logo, and same brown color for every pack) and many other countries are considering this measure as well [6].

An aspect of the cigarette pack that has received relatively little attention in the literature to date is the number of cigarettes it contains, that is, its size. While in the United States, for example, the average daily consumption of cigarettes has declined from a high of over 20 cigarettes per day in the 1960’s to 15 cigarettes per day in 2010 [7], the pack of 20 cigarettes remains the standard and minimum legal cigarette pack size. Smaller packs are also banned in most EU countries, a notable exception being the UK where cigarettes are commonly sold in packs of 10 or 20 units.

Departing from the widespread public health opinion that smaller packs should be banned on the grounds that they may encourage smoking initiation [8], one could argue that the availability of smaller packs could benefit some current smokers by helping them better regulate their consumption. A smaller pack size would help them ‘pre-commit’ to reducing their smoking rate. Pre-commitment approaches have been shown to be effective in reducing cigarette consumption and in other areas in studies of both humans [9, 10] and animals [11–13].

Almost no scientific study has investigated the impact of pack size on purchase intentions or consumption patterns. We are aware of only one study that investigates the role of pack size in cigarette demand and in which smokers are found to regulate their consumption according to available pack sizes [14]. Farrell and colleagues estimate the demand for cigarettes using a large national survey of smokers from the UK and find that smokers measure their cigarettes consumption in terms of packs rather than by counting the number of cigarettes [14]. They conclude that many smokers regulate their consumption through the use of small packs. A related literature has found that even though smokers could benefit from a lower price per cigarette by buying cartons, relatively few do so [15]. Similarly, Wertenboch finds that 90% of smokers bought a pack of cigarettes instead of a carton at their last purchase and 75% of these smokers indicated self-control as a reason for buying only a pack (Wertenbroch, 2001).

At the other end of the spectrum, several studies have investigated the use and availability of single cigarettes (i.e. “loosies”). While there is no clear evidence that loosies have an impact on youth smoking initiation, a study among currently smoking youth showed that 6–9% of 8^th^ to 12^th^-grade students bought less than a full pack of cigarettes at their last purchase [16]. In a study conducted in Mexico, Thrasher and colleagues [17] found some evidence that adult smokers are using them as a method to limit their consumption.

In this paper, we use insights from behavioral economics to study smokers’ demand for smaller cigarette packs. Specifically, we ask whether and if so why smaller cigarette packs would be appealing to adult smokers. We use a hypothetical purchase experiment between cigarette packs of different sizes and different corresponding price per cigarettes to uncover 1) whether current smokers would demand smaller packs if they were available on the market, 2) whether smokers would be willing to pay a price per unit premium to purchase smaller packs, and 3) what are the self-reported motivations for these choices. We start by presenting a conceptual framework that describes the determinants of pack size choice. We make a distinction between purchase of a stock of cigarettes (e.g., a cigarette pack) and marginal consumption (i.e., smoking the next cigarette). We discuss the role of price per cigarette, desire to quit smoking, self-control problem and the desire to pre-commit to reduce smoking. We then present results from an online survey investigating hypothetical demand for packs of different sizes and discuss the implications for tobacco use.

## Conceptual Framework

A majority of smokers regret their initial decision to smoke which is often made when they are young and also express a current desire to quit. This generates demand for both smoking and smoking cessation therapies- two conflicting demands [18, 19]. Further studies have found that smokers tend to support regulations that may help them quit by making it more difficult or more expensive to smoke (see e.g. [20, 21]). However, quitting is difficult and a desire to quit does not commonly translate into successful cessation. For example, in 2010, the CDC found that 67% of US smokers wanted to quit, whereas only approximately 5% of smokers successfully quit each year [7, 22]. These observations are inconsistent, or at least difficult to explain, with the neoclassical model of consumer choice. In contrast, behavioral economic models address such inconsistencies directly by including insights from psychology, focusing on, for example, self-control problems, time-inconsistent preferences and smoking cues. Many of these models focus on decisions involving addictive goods such as cigarettes [23–26] and allow for smoking ‘mistakes’ and differences between planned and actual consumption. Most models also predict that if time‐inconsistent agents (those with problems of self-control or self-regulation) anticipate their own mistakes (that is, they recognize their self‐control problems and plan ahead—they are ‘sophisticated’ [27–29]), they should have a demand for pre-commitment devices that can prevent them from making the wrong decisions in the future [9].

Here, we propose a simple framework that draws from this literature and that makes the distinction between planned consumption (e.g., smoking only 10 cigarettes today) and actual higher consumption due to the inability to resist temptation (see e.g. [30, 31]). More formally, we conceptualize cigarette consumption as a two-step decision-making process. First, we assume a forward-looking agent who decides how much she would like to smoke in the next period. Then, the agent may depart from her initial consumption objective if she is unable to resist to social, environmental and/or physiological cues over the consumption period. Consumption self-regulation is difficult for smokers due to the biology and psychology of addiction; physiological cues, such as craving for cigarettes; or visual cues via cigarette packaging. These can make the product especially tempting and may lead the smoker to put a disproportionate weight on present utility as opposed to long term negative consequences; therefore leading the smoker to consume more than planned [23, 26, 30]. In addition, once a pack is purchased, the transactional cost of each additional cigarette is low, i.e. the marginal cost of the next cigarette is zero in that the price of the pack is a sunk cost.

For simplicity, we assume that the forward-looking agent chooses, subject to a budget constraint, between a standard pack of 20 cigarettes, a smaller pack of 10 cigarettes at a per unit price premium, and a larger pack of 30 cigarettes at a discounted price per unit. For smokers whose budget constraint is binding, the small pack may be the only affordable option, especially if current market price is high, perhaps in part due to taxation. Also, occasional smokers may simply prefer the small pack because it better reflects their current consumption patterns. A smoker whose budget constraint is not binding is more likely to take advantage of the per cigarette discount associated with the larger pack, ceteris paribus. However, many smokers buy their cigarettes by the pack, although they could purchase cartons and benefit from quantity discounts [15]. These smokers may be acting strategically by rationing their purchase of cigarettes and therefore limiting their consumption opportunities [31] (we note that the high out-of-pocket up-front cost of a carton could partly explain this purchase patterns as well). Such a mechanism is likely to be valued by “sophisticated” smokers who both have a self-control problem and are aware of their problem. In other words, “sophisticated” smokers have an underlying demand for pre-commitment mechanisms and, in our context, the smaller pack of 10 cigarettes could serve as a commitment device to smoke less. These smokers may use a rationing rule such as “never buy more cigarettes than 10 units at a time”, yielding that consumption at higher rates only occurs at the expense of incurring additional transaction costs [31].

We propose several explanations of why buying a smaller pack may help smokers consume at levels that better reflect long-term preferences. While the marginal transactional and monetary cost of each cigarette is close to zero when a pack is within easy reach (e.g., in the pocket), the marginal cost of consumption sharply rises when the end of the pack is reached due to the time and inconvenience of having to buy another pack. With a small pack, smokers will run out of cigarettes more often. In addition, the extra time that it takes to replete the stock of cigarettes can serve as a cooling off period encouraging transition from impulsive (smoke immediately) to deliberative (“I want to quit”) decision-making [32]. Additionally, for the forward-looking smoker, the small pack may remind the smoker to pace smoking more slowly. As an analogy, evidence suggests that a smaller serving size can reduce food consumption. In a series of experiments, researchers have found that a larger container (bag, bowl or plate) can increase the amount of food consumed, often without the individual recognizing the increased consumption (see e.g., [33, 34]).

## Methods

Our primary objective was to examine smokers’ hypothetical demand for cigarette packs of various sizes. Because Federal regulations require cigarettes to be sold in packs of 20 units, we were unable to pursue a field experiment or to rely on actual purchase behavior in the US. Following previous studies that used choice experiments to analyze smoking decisions (see e.g., [35–43]), we relied on hypothetical purchases which have been shown to be reliable and valid in previous studies of cigarette and alcohol demand [44–46]. We designed an online experiment to examine: 1) the demand for standard (20 units), smaller (10 units), and larger (30 units) packs; and conditional on the initial choice of pack size: 2) the willingness to pay for a smaller (and larger) pack size; 3) the motivation for selection of the pack size; and 4) characteristics of smokers including their desire to quit and their self-reported degree of self-control.

Participants were recruited from the Yale School of Management eLab (elab.som.edu). This website offers a platform to post online surveys and experiments. A sample of adults is registered to the platform and receives regular notifications when new surveys are available. As compensation, respondents participate in a lottery to win an Amazon gift certificate. Our survey was advertised as a study on smoking habits restricted to adult smokers and was approved by Yale University’s Human Subjects Expedited Review Committee (protocol number: 1201009608). Participants provided their written informed consent before completing the online survey. Between March and April 2012, 868 respondents completed the survey. We restricted the sample to those who correctly answered a quality-control question (at the end of the survey, respondents were asked to write a specific word in a textbox as a validity check. Responses of those who did not read/follow the instructions were considered as invalid)(N = 661) and removed respondents with missing values on pack choice, price, and degree of self-control (N = 68), resulting in an analytic sample of 593 adult smokers (S1 Dataset).

### Survey

**Fig 1:**
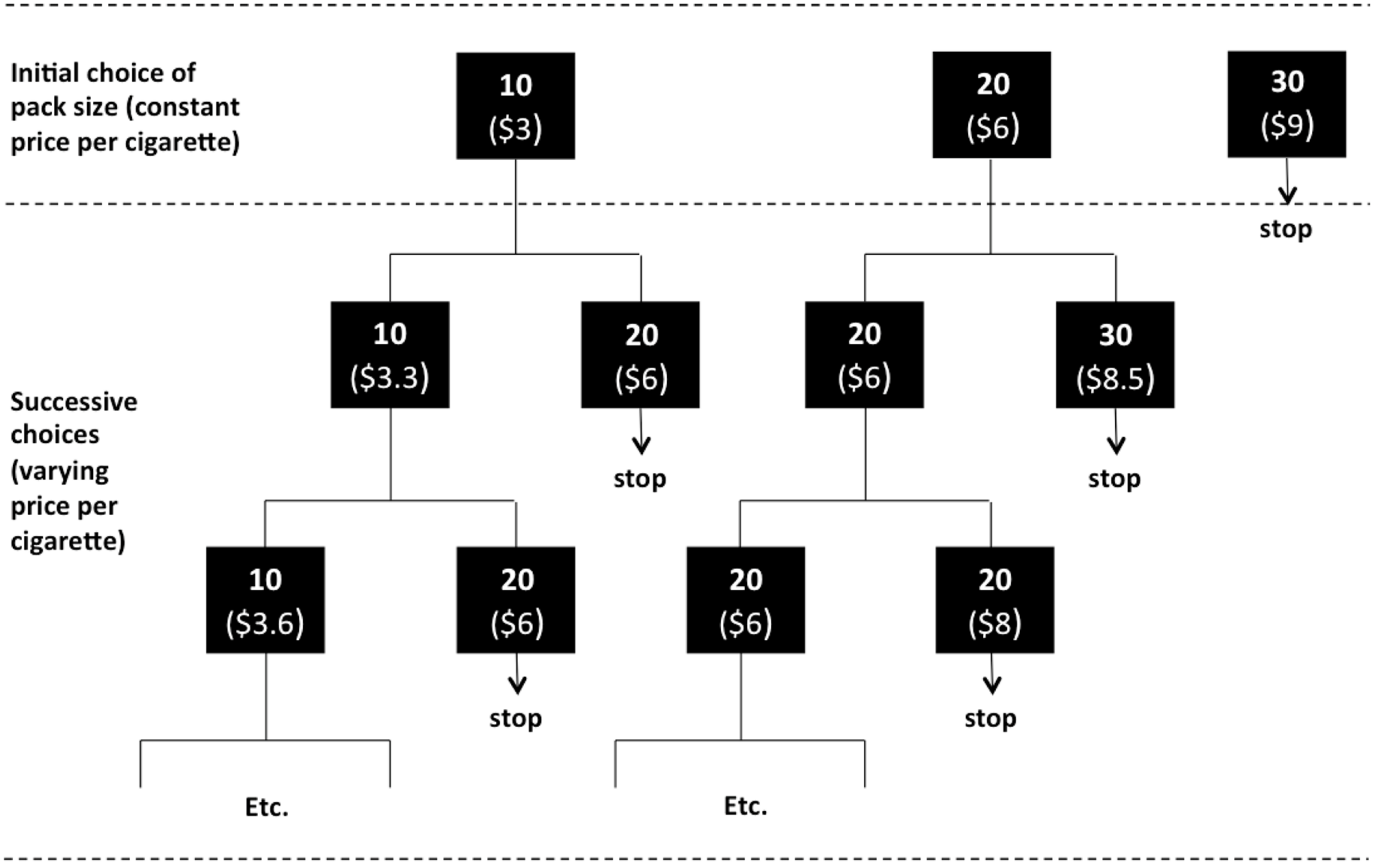
Structure of the choice experiment.

The survey consisted of two parts. First, respondents were asked to imagine going to their usual retailer to buy a pack of cigarettes and were given the hypothetical opportunity to choose between a pack of 10, 20 or 30 cigarettes. The price per cigarette was held constant across pack-sizes and was individualized using the respondents’ report of the price that they typically paid per pack. This was done to account for the important heterogeneity in prices across states and individuals—and to make the choice task more realistic. The second part of the experiment consists of a series of choices between various combinations of pack sizes and prices conditional on the initial choice made by respondents, as summarized in Fig 1. Specifically, if the respondent initially picked the pack of 10 cigarettes, she was then presented with successive choices between the standard pack (20 cigarettes at her reported price) and a pack of 10 cigarettes (at half of her reported 20-pack price) with a gradually increasing price of the 10-pack, reflecting an increasing price per unit (i.e., a “price premium”). The respondent was presented with price increases until she selected the pack of 20 cigarettes. This bidding process allowed retrieval of her maximum willingness-to-pay (or “per-cigarette premium”) for purchasing a small pack. Similarly, if the respondent initially picked the pack of 20 cigarettes, the successive choices consisted in the comparison of the standard pack and a pack of 30 cigarettes with a gradually decreasing price per unit (i.e. larger discount), until the respondent finally picked the large pack. This allowed us to quantify the per-cigarette discount needed for smokers to prefer to buy in larger quantities. Finally, if the respondent selected the pack of 30 as her initial choice, no further choices were presented. We then asked follow-up questions to better understand respondents’ choice of pack size.

We collected detailed information on current cigarette consumption and smoking history. Specifically, we asked respondents the price that they usually pay for a pack of cigarette (as mentioned above), the frequency at which they make their purchase and if they would like to quit smoking. We also asked a question regarding likely future smoking status and assessed the level of addiction using time to the first cigarette of the day [47]. Finally, our survey included a series of questions aimed at measuring respondents’ level of self-control. We used a subset of three questions most related to tobacco use from the brief version of the Self-Control Scale (SCS), developed by Tangney and colleagues [48]. Specifically, we use the items “I am good at resisting temptation”, “I refuse things that are bad for me”, and “I have hard time breaking bad habits” that are rated on a 6-point scale (from 0 “not at all” to 5 “very much”). The information from all three items was summarized into a single self-control index by summing all items (the last item was introduced with a negative sign; the score therefore ranges from -5 to 10). Finally, we collected information on age, gender, marital status, education, income, and ethnicity.

### Analyses

We first focus on respondents’ initial choice of pack size. We assume that there is a latent underlying factor that reflects preferences for cigarette pack size and therefore use an ordered probit specification. Alternative options include multinomial choice models but such models ignore the ordinal nature of the dependent variable, a key aspect of our empirical analysis. We assume a latent variable s* that reflects individual i’s preference for cigarette pack size:

$$s_{i}^{*}=\alpha p_{i}\;+\;\beta sc_{i}\;+\gamma quit_{i}+\text{ x}\prime \mu +{\text{e}}_{i}$$ (1)

where ‘p’ is the price the respondent currently pays for her cigarettes, ‘sc’ is a dummy variable that takes the value one for respondents with a high (above median) self-control score, ‘quit’ indicates whether the respondent expressed a desire to quit smoking and ‘x’ is a vector of individual characteristics. The vector x includes current consumption level (non-daily smoker, 0–5 cigarettes per day, 5–10 per day, 10–20 per day, 20+ per day), the frequency at which smokers purchase their cigarettes, a dummy for higher education, income, a proxy for addiction, and standard demographics (i.e., gender, age, marital status, ethnicity). To investigate potential non-linear impact of price we re-estimate the same models by including dummies reflecting the quartiles of price currently paid. We then focused our analysis on smokers who expressed a desire to quit (i.e. who answered “yes” to the question “would you like to quit smoking?” i.e. 230 individuals, or 39% of the sample) and estimated the same models in two subgroups, based on the answer to a question on future smoking status (i.e., “Will you be a smoker in five years?”), to distinguish between “optimistic quitters” and “pessimistic quitters”

In the next step we exploited responses from the choice experiment to obtain estimates of the distribution of price premium and price discount in the subgroups determined by pack size choices. Among smokers who initially picked the small pack, the price premium was estimated from the price at which the respondent switched to prefer the regular pack. For instance, if the regular 20-pack costs $5 (i.e. 25 cents per cigarettes) and the highest price at which the respondent still preferred the small 10-pack was $3 (i.e. 30 cents per cigarette), the resulting price premium was 5 cents per cigarette (equivalent to 20% of the initial price per unit). Among smokers who initially picked the regular pack, we computed the minimum price discount they needed to switch to the large pack. For instance, if the regular 20-pack cost $5 (25 cents/cigarette) and the respondent chooses the large 30-pack when it cost $6 (20 cents/ cigarette), the corresponding price discount was 5 cents per cigarette (20% of the initial price per cigarette).

## Results

The summary statistics for our analytic sample are presented in Table 1. We observe that smokers who prefer the small cigarette pack are less likely to be daily smokers, smoke less per day and are less addicted. Also, they are more likely to want to quit and are less likely to think they will be smoking in the future. In terms of other individual characteristics, smokers who prefer the small cigarette pack are younger and have higher education, income, and self-control.

**Table 1:** Summary statistics: entire sample and by initial pack size selected.

		By pack size initially chosen			T-tests (p-values reported)	
	Entire sample	Chose pack of 10	Chose pack of 20	Chose pack of 30	10 vs. 20	30 vs. 20
	(1)	(2)	(3)	(4)	(5)	(6)
**N (%)**	**593**	**213 (36%)**	**224 (38%)**	**156 (26%)**		
**Panel A. Individual characteristics**						
Male	0.47	0.51	0.39	0.51	0.013	0.021
Age						
*18–34*	0.55	0.66	0.47	0.52	0.000	0.334
*35–54*	0.32	0.26	0.35	0.35	0.042	0.995
*55+*	0.13	0.08	0.18	0.13	0.001	0.186
Married	0.43	0.39	0.42	0.51	0.525	0.073
Higher education	0.48	0.59	0.41	0.45	0.000	0.411
Household income						
*< 35*,*000$*	0.34	0.33	0.38	0.31	0.311	0.176
*35–65*,*000$*	0.38	0.33	0.38	0.46	0.364	0.119
*>65*,*000$*	0.28	0.33	0.25	0.24	0.043	0.776
Self-control index	1.70	2.23	1.38	1.40	0.000	0.925
**Panel B. Smoking behavior**						
Price per pack in $ (sd)	6.04 (3.07)	6.35 (3.27)	5.95 (2.75)	5.80 (3.20)	0.162	0.559
Daily consumption						
*Non daily smoker*	0.22	0.40	0.14	0.09	0.000	0.119
*0–5*	0.14	0.17	0.10	0.13	0.031	0.340
*5–10*	0.20	0.14	0.26	0.18	0.002	0.069
*10–20*	0.19	0.17	0.17	0.24	0.991	0.098
*20+*	0.25	0.11	0.32	0.35	0.000	0.528
Wants to quit smoking	0.39	0.42	0.41	0.32	0.806	0.089
First cigarette <30 min.	0.54	0.38	0.64	0.63	0.000	0.771
Smoker in 5 years	0.48	0.34	0.50	0.65	0.001	0.002

Note: P-values of two-sided t-tests reported in the two last columns

Results from the ordered probit model are shown in Table 2. Column 1 presents results in the full sample. We observe a clear association between current consumption levels and preference for pack size. Daily smokers are consistently and significantly more likely to select a larger pack size than non-daily smokers (the reference category), and the preference for a larger pack increases monotonically with daily consumption levels. Results also show that both high self-control and willingness to quit are significantly associated with a preference for a smaller pack. Finally, while a higher market price (i.e. living in a region with more expensive cigarettes) yields a tendency to choose a smaller pack, the coefficient is not statistically significant despite fairly high geographic price variation. This may be related to the fact that we are relying on self-reported prices, which may be endogenous to preferences for pack size and therefore could bias the results. The non-linear impact of price is explored in column 2. Results show that smokers who are in the top quartile of price paid (e.g. who live in high tax states), tend to favor smaller packs. Columns 3 and 4 in Table 2 present the results in two subgroups of smokers who express a desire to quit, those who are: optimistic (i.e., think they will not smoke in 5 years) versus pessimistic (think they will smoke in 5 years) quitters. Results indicate that self-control is associated with preference for a smaller pack only among “optimistic” quitters. Also, we observe that price plays a significant role in pack size choice only among pessimistic quitters. Price may not play as important of a role for the optimistic quitters because they likely anticipate low future consumption. This would be consistent with models of rational addiction [49].

**Table 2:** Choice of cigarette pack size.

			Smokers who would like to quit	
	Full sample (1)	Full sample (2)	Optimistic quitters (3)	Pessimistic quitters (4)
Price (linear)	-0.013 (0.162)	-	-0.005 (0.042)	-0.222** (0.103)
Price (ref: 1^st^ quartile)				
*2* ^*nd*^ *quartile*	-	-0.123 (0.131)		
*3* ^*rd*^ *quartile*	-	-0.121 (0.142)		
*4* ^*th*^ *quartile*	-	-0.233* (0.103)		
Wants to quit smoking	-0.270*** (0.103)	-0.268*** (0.103)	-	-
High self-control index	-0.193* (0.104)	-0.198* (0.105)	-0.474** (0.202)	0.045 (0.492)
Daily consumption level (ref: non-daily smoker)				
*<5*	0.585*** (0.177)	0.587*** (0.177)	0.339 (0.334)	-0.832 (1.298)
*5–10*	0.743*** (0.181)	0.738*** (0.180)	1.100*** (0.367)	-0.546 (0.686)
*10–20*	0.801*** (0.191)	0.793*** (0.191)	1.149*** (0.410)	0.057 (0.654)
*>20*	1.053*** (0.193)	1.042*** (0.193)	1.769*** (0.413)	0.243 (0.742)
Purchase frequency (ref: daily)				
*Weekly*	0.200 (0.139)	0.196 (0.141)	1.067*** (0.340)	0.097 (0.572)
*Monthly*	0.087 (0.155)	0.080 (0.155)	1.159*** (0.372)	-0.313 (0.664)
First cigarette <30 min	0.133 (0.111)	0.121 (0.110)	0.227 (0.248)	1.061 (0.780)
Male	0.110 (0.104)	0.107 (0.104)	-0.128 (0.213)	-0.079 (0.406)
Age (ref: 18–34)				
*35–54*	-0.059 (0.116)	-0.060 (0.116)	-0.576** (0.246)	0.311 (0.514)
*55+*	-0.026 (0.160)	-0.037 (0.161)	-0.486 (0.306)	0.098 (0.657)
Married	0.071 (0.104)	0.068 (0.104)	0.063 (0.204)	1.062 (0.473)
White	0.155 (0.123)	0.154 (0.123)	0.147 (0.254)	0.022 (0.573)
Higher education	-0.143 (0.106)	-0.147 (0.106)	-0.072 (0.220)	-0.323 (0.441)
Household income (ref: <$35,000)				
*35–65*,*000$*	0.081 (0.121)	0.085 (0.121)	0.157 (0.225)	-0.698 (0.523)
*>65*,*000$*	-0.150 (0.134)	-0.140 (0.134)	0.238 (0.255)	-0.417 (0.651)
**N**	**593**	**593**	**176**	**54**

Ordered probit coefficients reported. Standard errors in parentheses. Optimistic smokers (column 2) are those who would like to quit and think they will no longer smoke in 5 years. Pessimistic smokers (column 3) are those who would like to quit and think they will still smoke in 5 years.

*p<0.10

**p<0.05

*** p<0.01.

Table 3 summarizes the reasons respondents cited for their choice of pack size (respondents could select more than one reason) in the full sample and in high (>$65,000) and low (<$35,000) income groups. Importantly, over 70% of smokers who picked the small packs said they did so in order to better regulate their consumption while almost 40% said they did so because it was cheaper. Almost 80% of smokers who picked the standard size as their preferred option did so “because they are used to it”, providing evidence of a possible status quo bias. While do not observe large differences by income group, low income smokers tend to report more frequently consumption regulation as the reason to choose the small pack. The remaining analyses focused on responses to the successive choice tasks with varying price per cigarette. Specifically we focused on the respondents who initially either picked the small or the conventional pack. As described above, we were able to derive estimates of the minimum price at which they decided to change their initial pack size option. Results suggest that two-thirds of smokers who initially selected the small pack were willing-to-pay at least a 10% premium to satisfy their preference for the smaller pack, resulting in an average premium of 7.4 cent per cigarette (equivalent to an average 22% premium). Table 4 summarizes these findings. Of note, a non-negligible proportion of respondents (9%) were willing to pay a 100% premium to purchase the small pack whereas few respondents had a premium between 60% and 90%. While this may reflect strong preferences for the small pack, this might also be explained by a poor understanding of the choice task for these respondents.

**Table 3:** Reasons for pack size choice.

Reasons for choosing pack of 10	Full sample	Low income (<$35k)	High income (>$65k)
To limit my consumption	72.8%	78.6%	70.8%
Price	38.9%	37.1%	40.3%
Convenience (easier to carry)	26.7%	27.1%	25%
Other	1.9%	4.3%	0.0%
*N*	*213 (36%)*	*70*	*72*
**Reasons for choosing pack of 30**			
To never run out of stock	82.1%	87.5%	78.4%
It is better adapted to my consumption	32.7%	25%	32.4%
Other	2.6%	2.1%	2.7%
*N*	*156 (26%)*	*48*	*37*
**Reasons for choosing pack of 20**			
I am used to it	78.6%	75%	82.1%
It is the ideal size	34.4%	27.4%	42.9%
Other	6.7%	11.9%	1.8%
*N*	*224 (38%)*	*84*	*56*

Note: sums exceed 100% due to multiple answers

**Table 4:** Premium to purchase a small pack (smokers who chose a pack of 10 only).

Premium in % of current price per cigarette	N (%)
No premium	74 (34.7%)
10%	32 (15.0%)
20%	27 (12.7%)
30%	30 (14.1%)
40%	12 (5.6%)
50%	8 (3.8%)
60%	2 (0.9%)
70%	5 (2.3%)
80%	2 (0.9%)
90%	2 (0.9%)
100%	19 (8.9%)
**N**	**213**
*Average price per pack*	*$ 6*.*35*
*Average premium*	*7*.*4 cts /cigarette*

The average premium is obtained using information on actual price paid by respondents.

Finally we turn to the analysis of the choices made by smokers who initially chose the regular pack of 20. Specifically, their choices allowed us to examine the price discount they would need to switch to a larger pack. In Table 5, we see that about 30% of smokers would switch to a larger pack with only a 5% discount on the price per cigarette. The average discount needed to switch to a larger pack is of 4.7 cents per cigarettes, corresponding to an average discount of 10.1%. Looked at from another perspective, estimates of the discount required to switch to a larger pack provide an estimate of the maximum implicit willingness to pay for the smaller pack size (20 cigarettes) as compared to the larger (30 pack).

**Table 5:** Discount needed to switch to the large pack (smokers who chose a pack of 20 only).

Premium in % of current price per cigarette	N (%)
5%	56 (25%)
10%	33 (14.7%)
15%	35 (15.6%)
20%	30 (13.4%)
25%	18 (8.0%)
33%	18 (8.0%)
Does not switch	34(15.2%)
**N**	**224**
*Average price per pack*	*$ 5*.*95*
*Average discount*	*4*.*7 cts / cigarette*

The average discount is obtained using information on actual price paid by respondents.

## Discussion

The current standard cigarette pack size of 20 units is a convention, but it has never been demonstrated to be the optimal size from the perspective of the smoker or society at large. Given the decline in the average rate of smoking over the past few decades, it may be appropriate to revisit and research the pack size to reflect and reinforce the trend toward an overall reduction in cigarette consumption. To investigate this issue, we use insights from behavioral economics and analyze results of a hypothetical purchase experiment of various cigarette packs.

Our findings suggest that about one third of current smokers would be interested in buying a smaller pack of 10 cigarettes and that most of them report consumption regulation as their main reason for choosing a smaller pack. These smokers are willing to pay a premium for the relatively smaller pack, which is consistent with a demand for a pre-commitment device. Results from regression models show that preferences for pack sizes match current consumption of cigarettes. However, we also find that smokers who are interested in quitting and who have a higher degree of self-control have a preference for smaller packs.

The relationship between self-control and pack size preference is theoretically ambiguous. Those with higher self-control might have built their self-control through use of mechanisms that help them resist temptation; or they may believe that they do not need the smaller pack size as a pre-commitment device. Thus our finding that higher self-control is associated with demand for the smaller pack size is informative. Perhaps higher self-control reflects an ability to recognize self-regulating mechanisms. That a desire to quit smoking is also associated with selection of the smaller pack size is consistent with the demand for a pre-commitment device. Finally, by separately examining those who are optimistic versus pessimistic about their ability to quit, we find that price is significant only in the pessimistic group. The latter is consistent with the income effect predominating in those who expect to continue to smoke.

While our findings support the concept of a smaller pack size to help control smoking for some current smokers, we realize that a crucial issue is whether the smaller pack would induce youths to start smoking at a greater rate [50]. In fact the US, the Family Prevention and Tobacco Control Act recently gave the Food Drug Administration [51] the authority to regulate tobacco and the Act explicitly banned pack sizes smaller than 20 cigarettes primarily on the grounds that this could increase youth smoking. Similarly, following a report showing that packs of ten were the preferred option for three-quarters of smokers aged between 16–17 years, Ireland made the minimum pack size 20 in 2007. In a recent paper, Kotnowski and Hammond examined tobacco company documents that studied the likely impact of cigarette pack attributes on consumer perceptions and behaviors [8]. These documents suggest that small packs are attractive to young adults and that thin packs are valued for their convenience (i.e. easy to carry, fits in purse, etc.). However, there is little to no empirical evidence to confirm or reject this concern. We note though that only about 15 percent of youths under age 18 directly purchase their own cigarettes from stores or gas stations [52] and evidence is mixed on whether price affects youths demand for cigarettes (see e.g., [53, 54]). An analogy can be drawn between the sale of small cigarette packs and the sale of single cigarettes (or “loosies”) [16, 17] that could act as a gateway to regular smoking for youth. However, loosies are sold only illegally and in very specific settings, making the comparisons with small packs difficult. Thus the extent to which the smaller pack size would increase youth consumption is an open issue. Also further FDA regulations to deter access to cigarettes would pave the way for the use of the smaller pack size with less concern about youths starting to smoke. The market for regular tobacco cigarettes is changing with the rapid growth of e-cigarettes, and the change is most dramatic for youths. Currently in the US, for example, more youths smoke e-cigarettes than regular combustible cigarettes [55]. Of note, a regulation that would make 10-packs the only size available or requiring subpacks of five within a pack of twenty would be considered soft paternalism, as smokers would be still free to purchase cigarettes in any quantities.

Another concern is that the lower price of a smaller pack might appeal to low income smokers and make smoking look more affordable. However, due to fixed production and marketing costs, the price per cigarettes in small packs is likely to be higher, which according to the literature would reduce the demand for cigarettes. In other words, while the upfront cost for the small pack would be lower and could facilitate the maintenance of smoking habits, the spending would remain the same (or increase) for a given consumption level.

There are several strengths of this research including that that we produce some of the first experimental evidence on this topic. Also, the survey allows us to derive detailed information on preferences with relatively realistic choice tasks. However, the hypothetical nature of the choices is also a key limitation of this study. In addition, due to the data collection platform, the sample was not representative of the US population of smokers.

To conclude, there is no evidence to support the 20 cigarettes per pack as optimal; it represents only the status quo and thus should be rethought in the light of both declining cigarettes smoked per day and increasing regulations limiting smoking. It is instructional to note that cigarettes in the US used to be sold most commonly in packs of 25, but now 20 [56]. Smaller packs have been banned in Ireland and other countries but we do not think that these policies were supported by empirical evidence. This is why we think that this topic should be opened up to more research. While we provide some preliminary evidence that smaller packs may be beneficial to certain types of smokers, further research should be conducted to assess whether the smaller pack size should be considered in the arsenal of tobacco control policies to help current smokers quit. This could be done by using a field experiment or by exploiting regulatory changes in other countries.

## Supporting Information

S1 Dataset Dataset used for the analysis. (XLS)
